# Supplementary material for: Cystathionine-β-synthase is essential for AKT-induced senescence and suppresses the development of gastric cancers with PI3K/AKT activation
Source: eLife. 2022 Jun 27;11:e71929. doi: 10.7554/eLife.71929 (PMC9236611; doi:10.7554/eLife.71929)
Supplement: Figure 1—source data 1. — Raw images were acquired using the ChemiDoc system (Bio-Rad). [file elife-71929-fig1-data1.pdf]

# Figure 1-source data 1

Unedited immunoblots of Figure 1B.

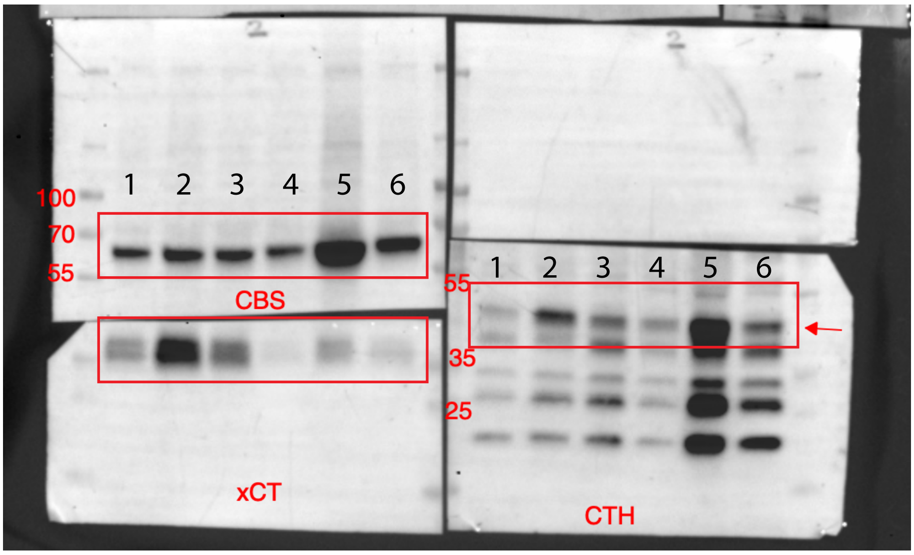


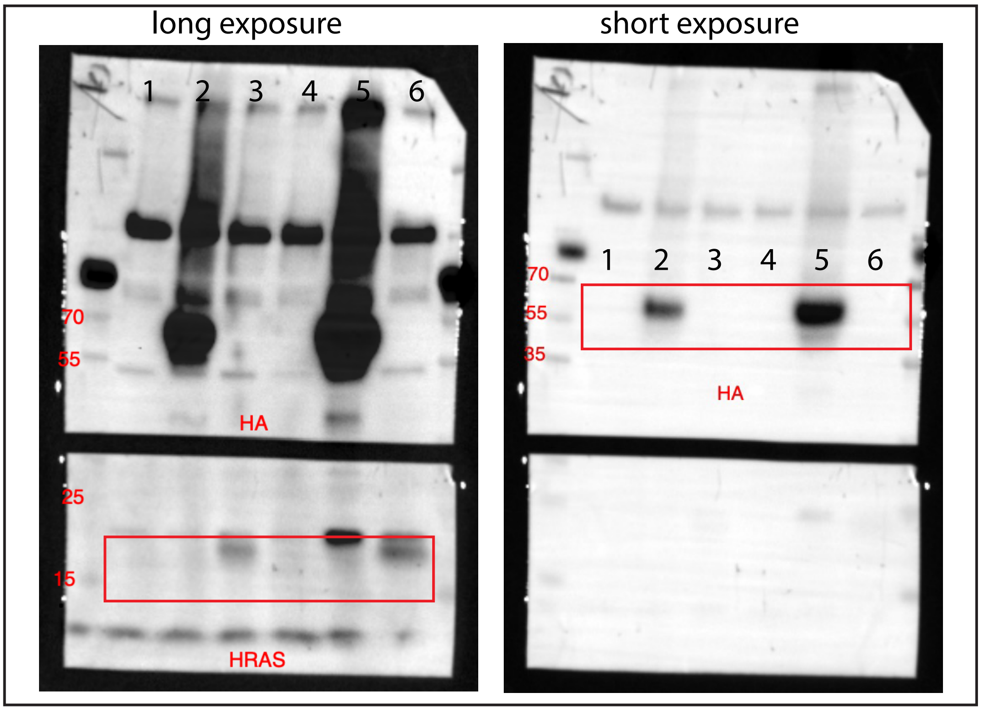


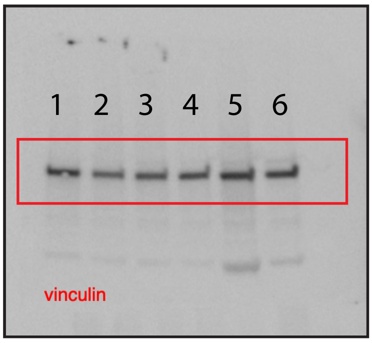


1. pBabe_FM
2.myrAKT1_FM
3. HRAS$^{G12V}$_FM
4. pBabe_Cys-free
5. myrAKT1_Cys-free
6. HRAS$^{G12V}$_Cys-free
