# Supplementary figures and images for: Cystathionine-β-synthase is essential for AKT-induced senescence and suppresses the development of gastric cancers with PI3K/AKT activation
Source: eLife. 2022 Jun 27;11:e71929. doi: 10.7554/eLife.71929 (PMC9236611; doi:10.7554/eLife.71929)

## Figure 1-figure supplement 1-source data 1

Unedited immunoblots of Figure 1-figure supplement 1B

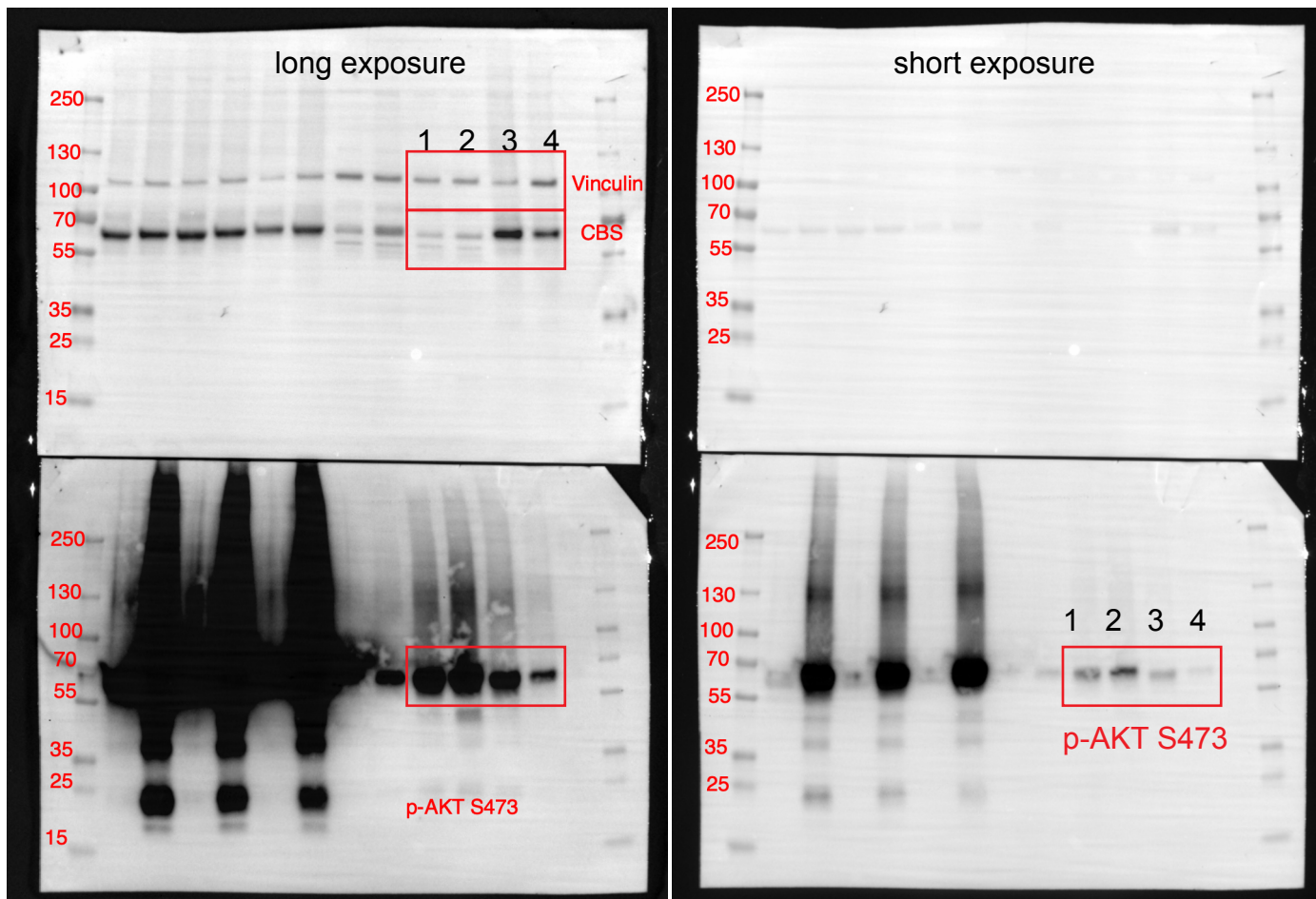

Supplement: Figure 1—figure supplement 1—source data 1. — Raw images were acquired using the ChemiDoc system (Bio-Rad). [file elife-71929-fig1-figsupp1-data1.pdf]

## Figure 2-source data 1

Unedited immunoblots of Figure 2A

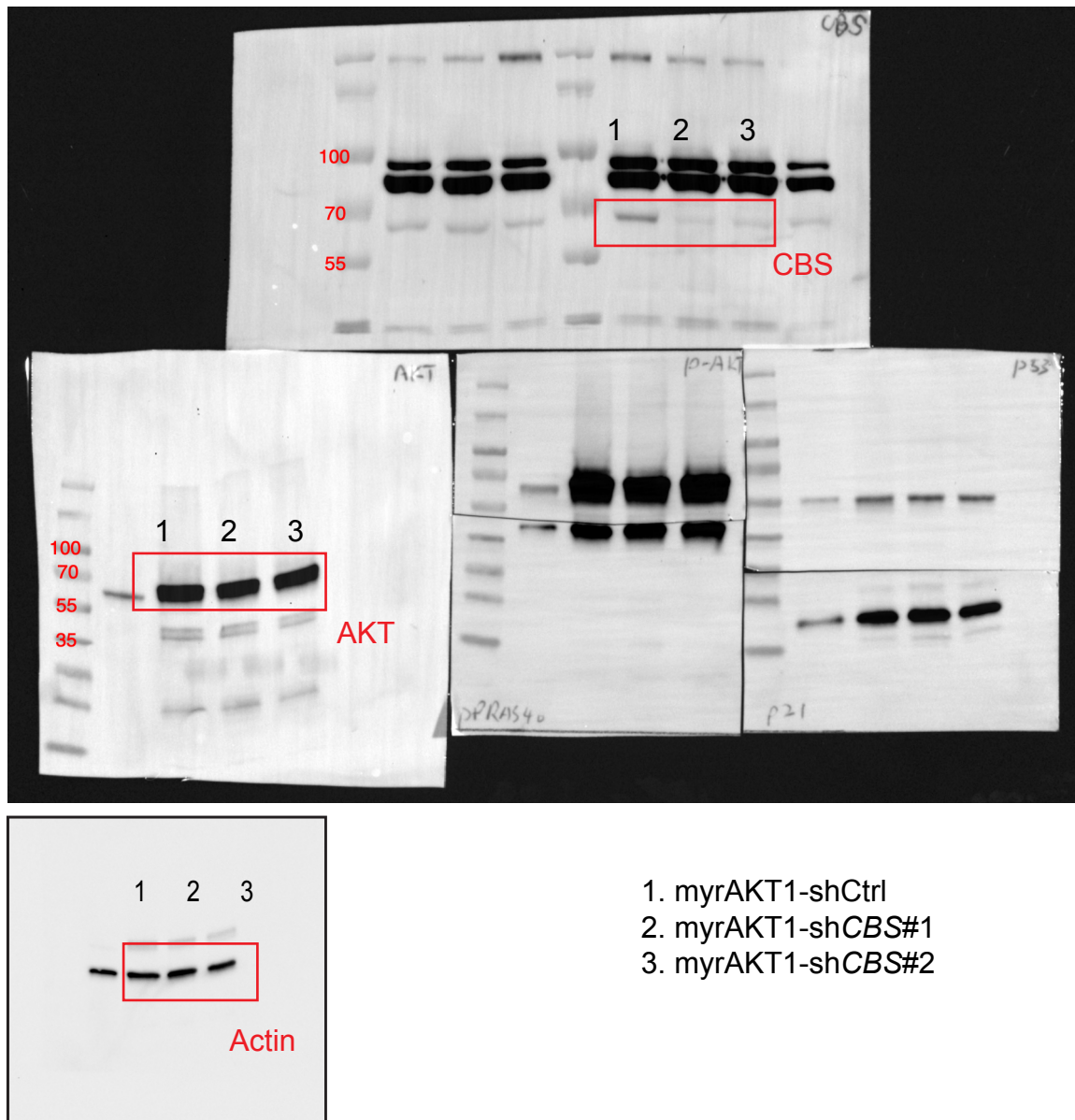

Supplement: Figure 2—source data 1. — Raw images were acquired using the ChemiDoc system (Bio-Rad). [file elife-71929-fig2-data1.pdf]

# Figure 2-source data 3

Unedited immunoblots of Figure 2F

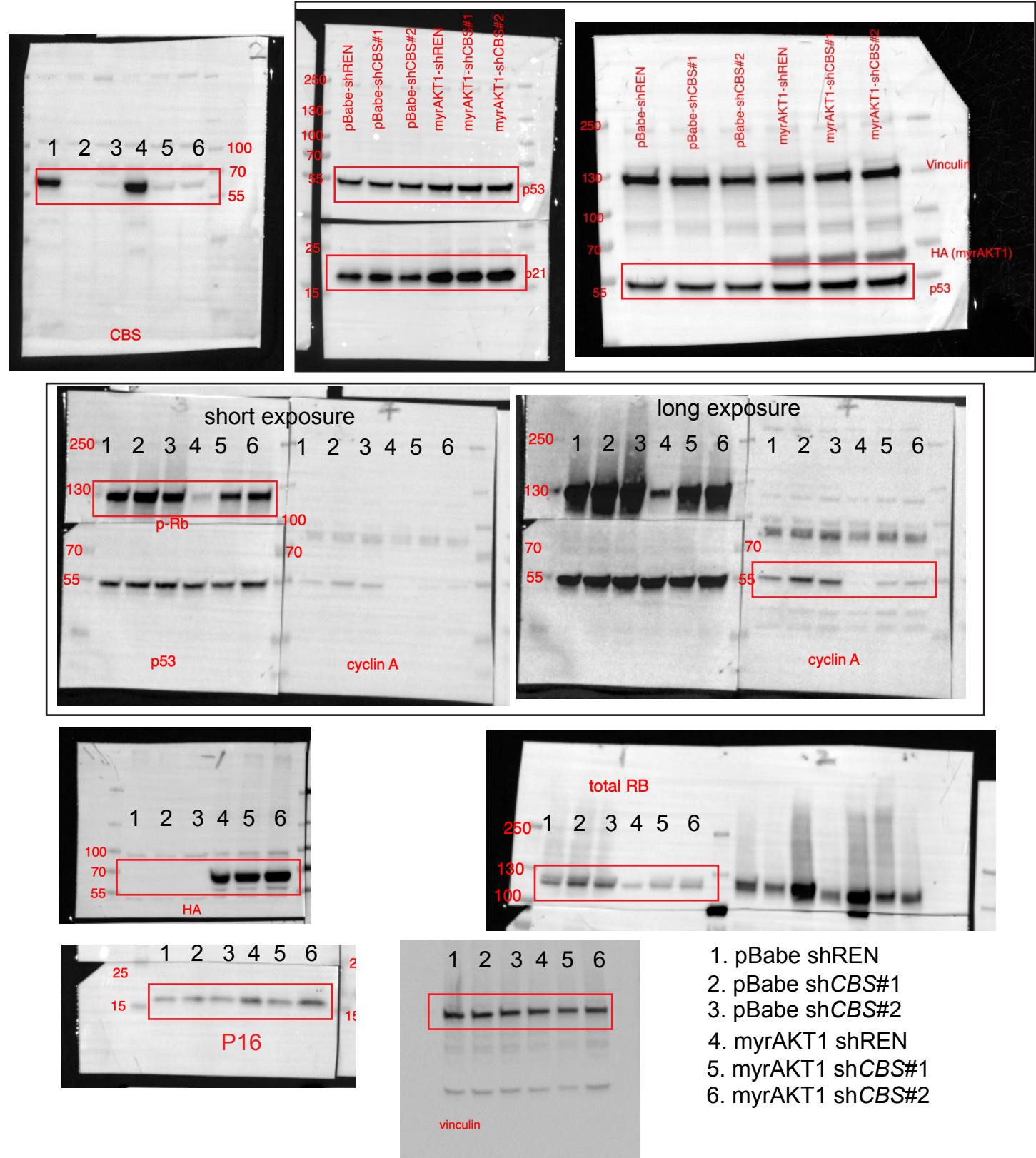

Supplement: Figure 2—source data 3. — Raw images were acquired using the ChemiDoc system (Bio-Rad). [file elife-71929-fig2-data3.pdf]

# Figure 2-figure supplement 1-source data 2

Unedited immunoblots of Figure 2-figure supplement 1E

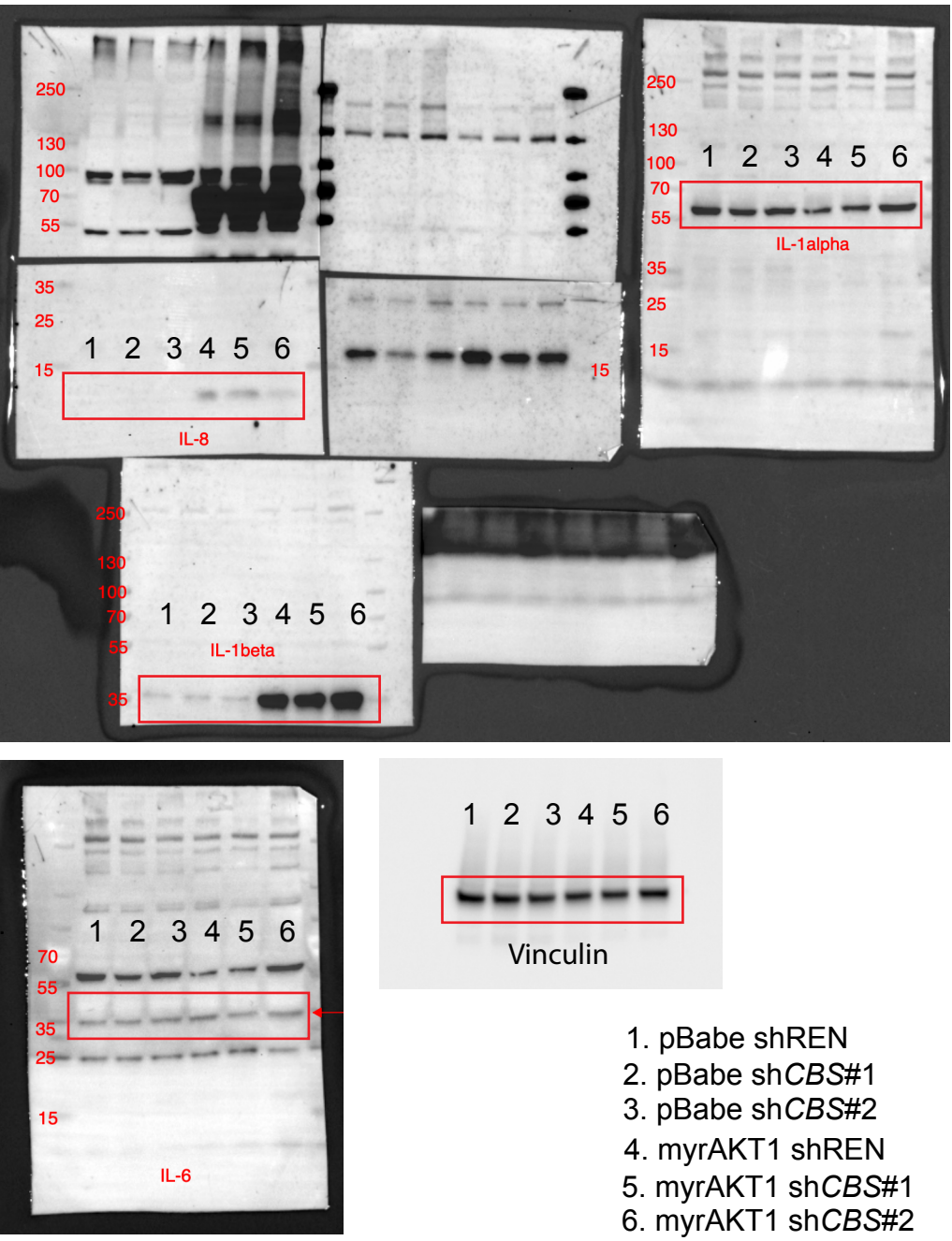

Supplement: Figure 2—figure supplement 1—source data 2. — Raw images were acquired using the ChemiDoc system (Bio-Rad). [file elife-71929-fig2-figsupp1-data2.pdf]

# Figure 4-source data 1

Unedited immunoblots of Figure 4C.

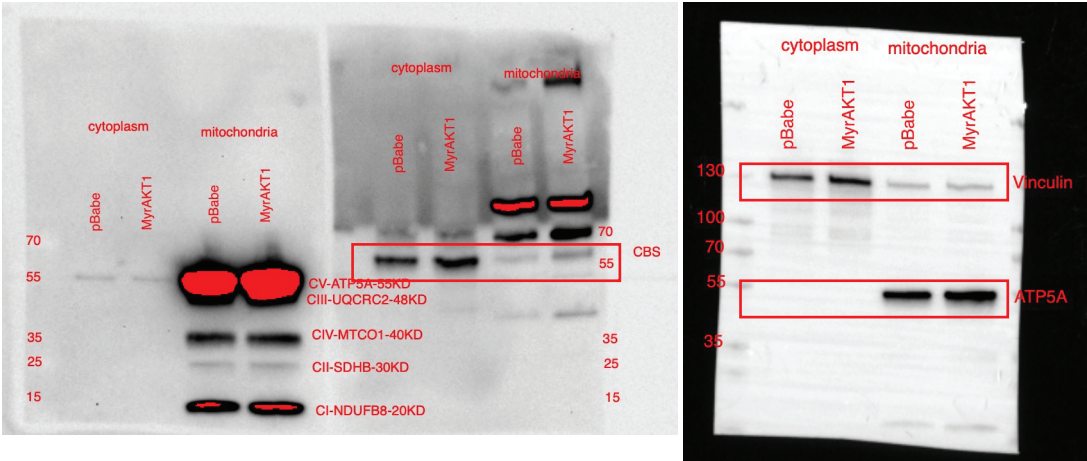

Supplement: Figure 4—source data 1. — Raw images were acquired using the ChemiDoc system (Bio-Rad). [file elife-71929-fig4-data1.pdf]

# Figure 4-source data 2

Unedited immunoblots of Figure 4D.

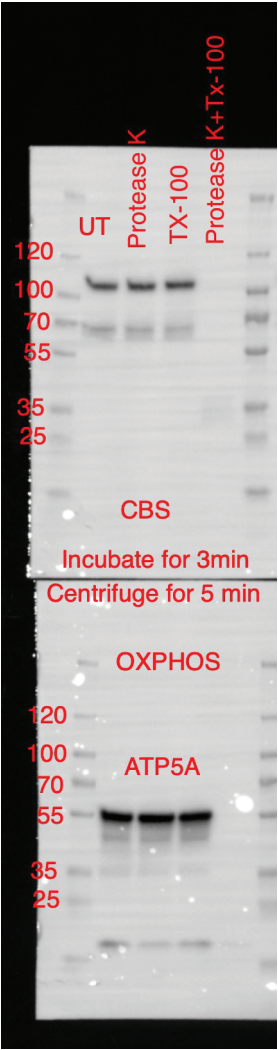

Supplement: Figure 4—source data 2. — Raw images were acquired using the ChemiDoc system (Bio-Rad). [file elife-71929-fig4-data2.pdf]

# Figure 4-supplement 1-source data 1

Unedited immunoblots of Figure 4-figure supplement 1A

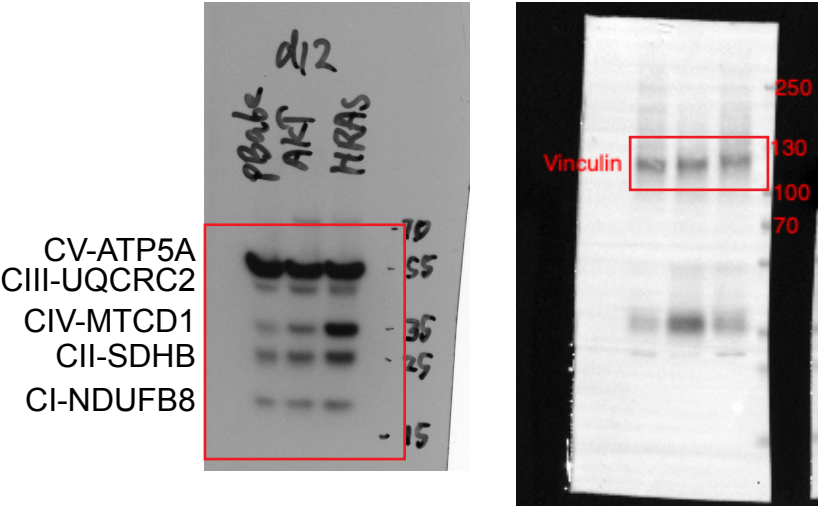

Supplement: Figure 4—figure supplement 1—source data 1. — Raw images were acquired using the ChemiDoc system (Bio-Rad). [file elife-71929-fig4-figsupp1-data1.pdf]

# Figure 6-source data 1

Unedited immunoblots of Figure 6F

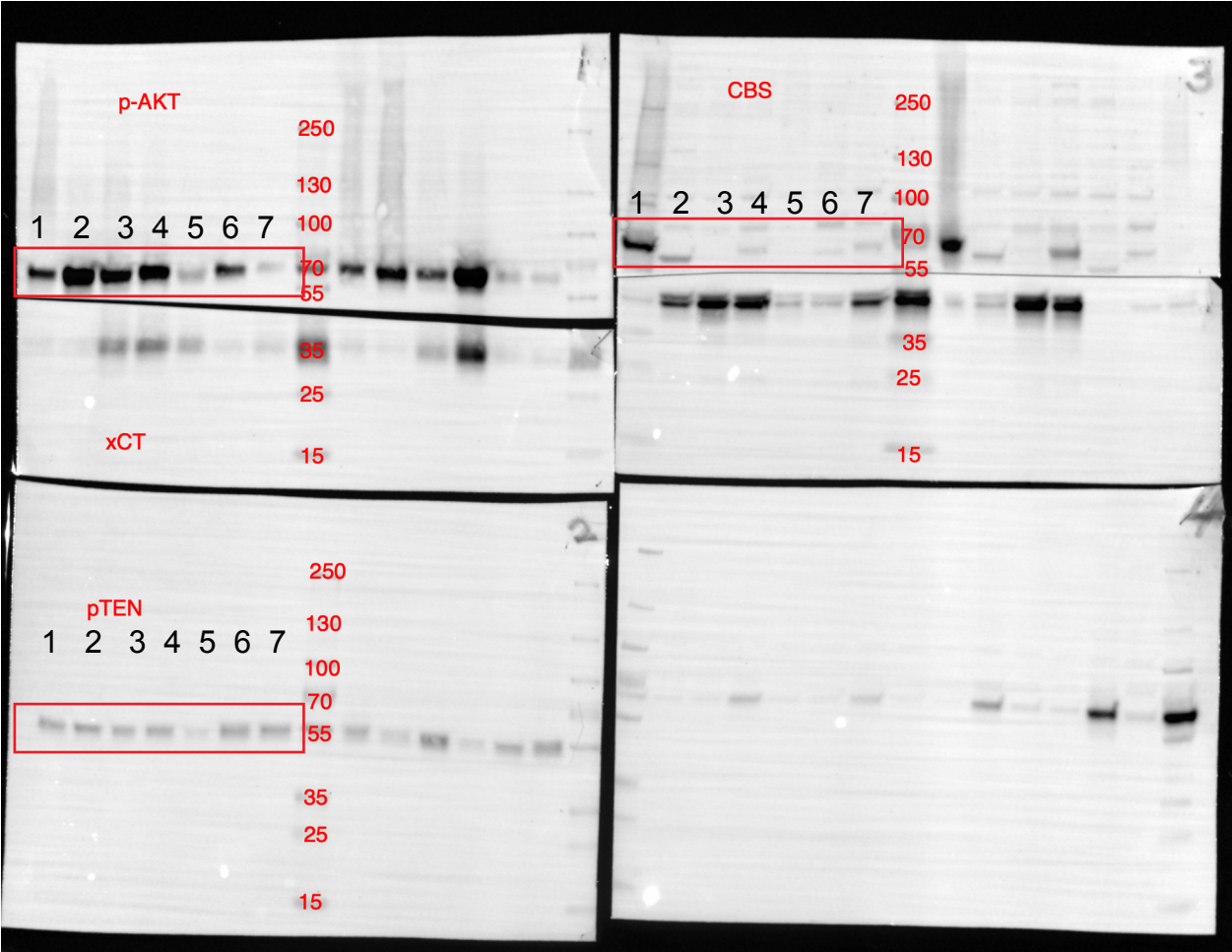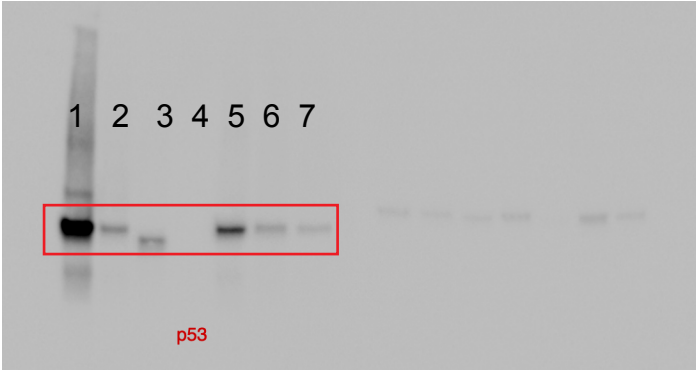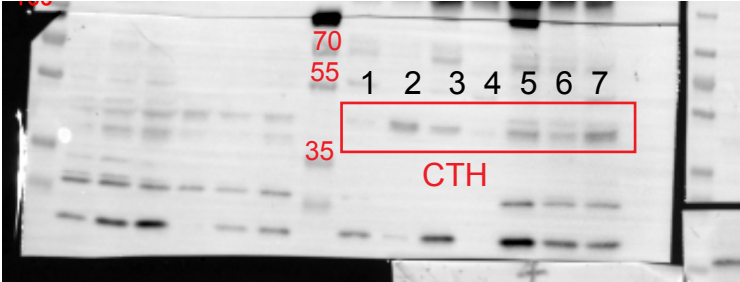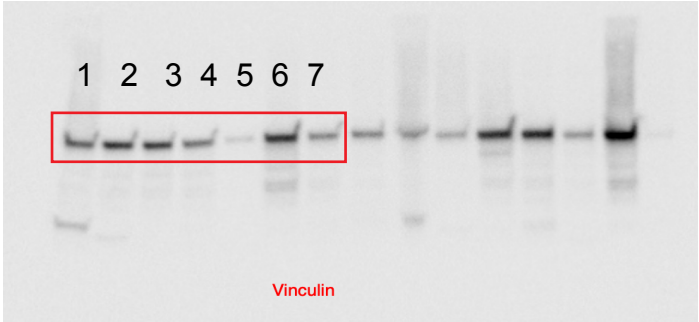

- 1. GES-1
- 2. AGS
- 3. Hs746T
- 4. KATOIII
- 5. NCI-N87
- 6. NCI-SNU1
- 7. NCI-SNU5

Supplement: Figure 6—source data 1. — Raw images were acquired using the ChemiDoc system (Bio-Rad). [file elife-71929-fig6-data1.pdf]

## Figure 6-figure supplement 1-source data 1

Unedited gel image of Figure 6-figure supplement 1D

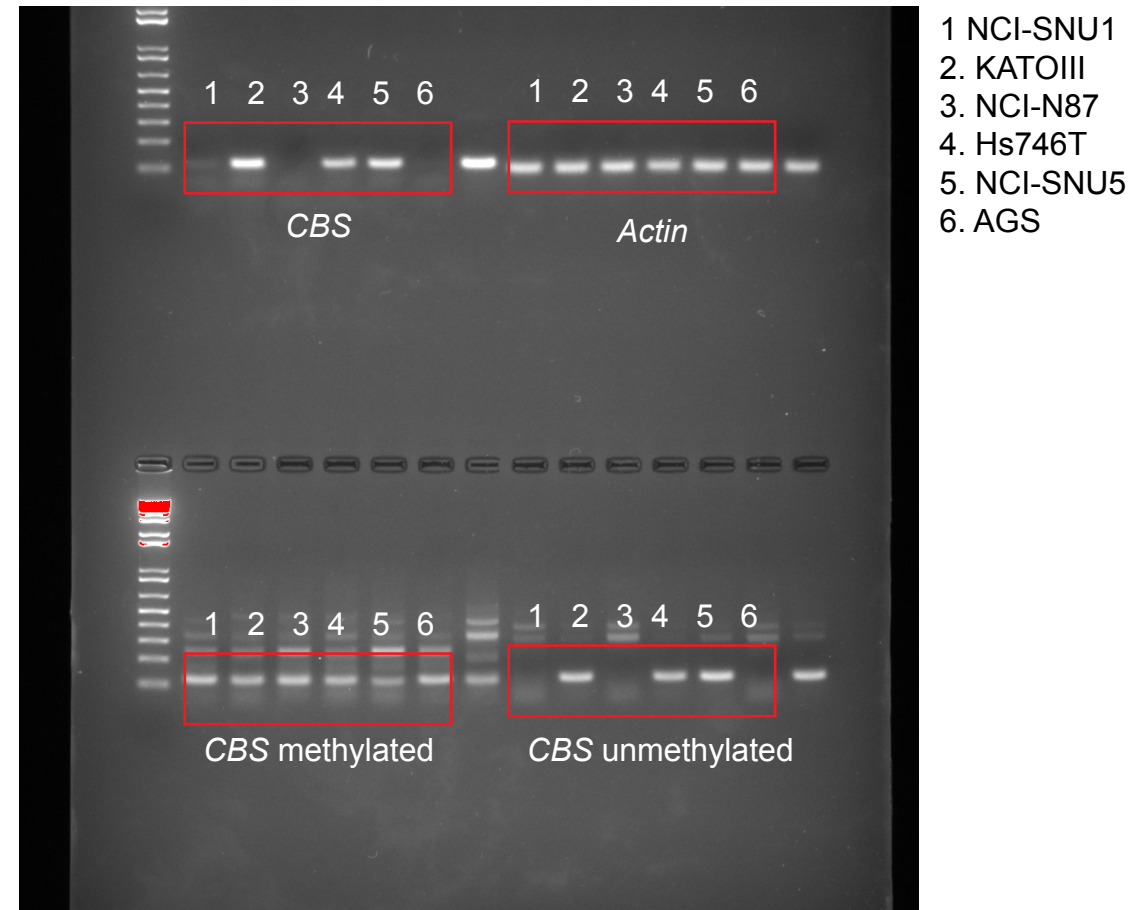

Supplement: Figure 6—figure supplement 1—source data 1. — Raw images were acquired using the ChemiDoc system (Bio-Rad). [file elife-71929-fig6-figsupp1-data1.pdf]

## Figure 7-source data 1

Unedited immunoblots of Figure 7A

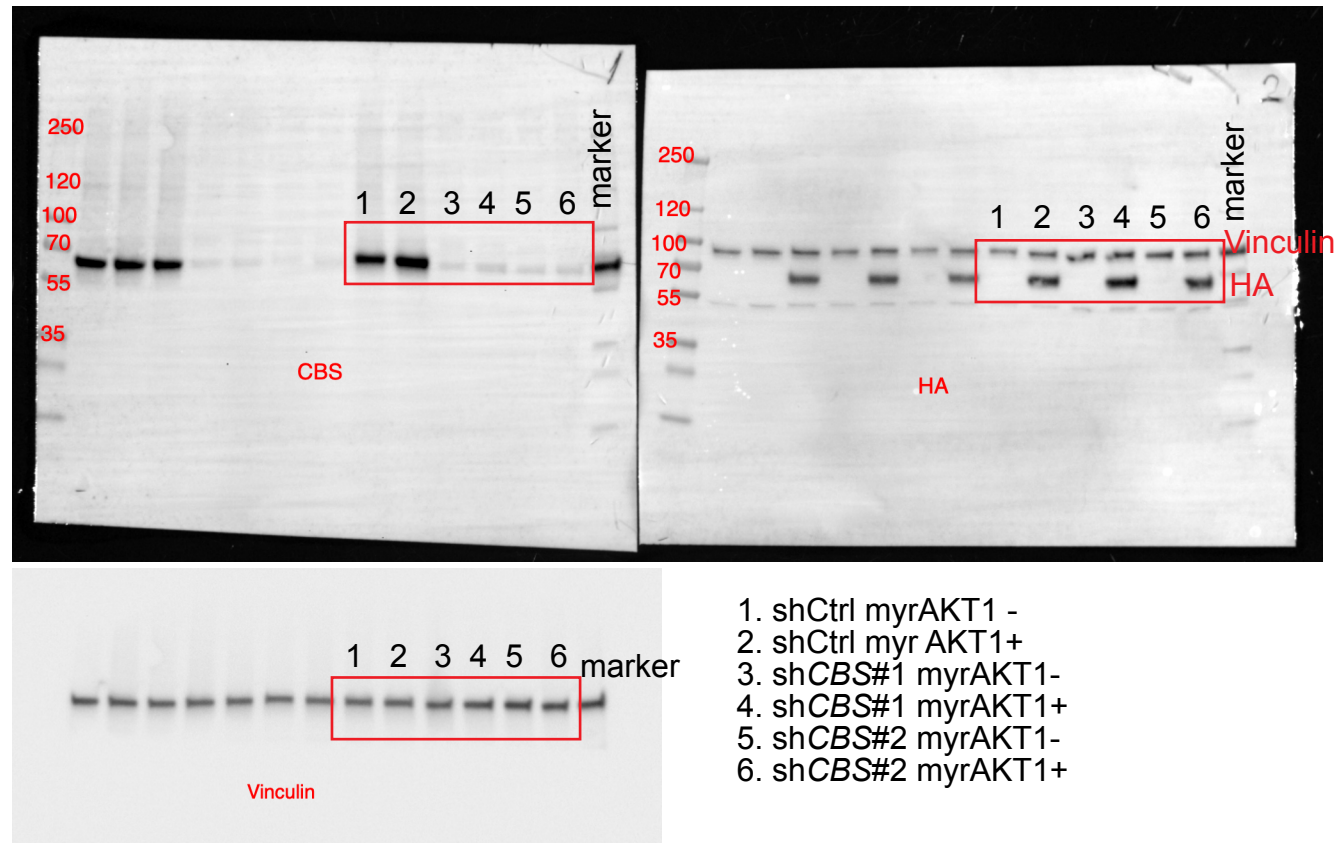

Supplement: Figure 7—source data 1. — Raw images were acquired using the ChemiDoc system (Bio-Rad). [file elife-71929-fig7-data1.pdf]

## Figure 7-source data 3

Unedited immunoblots of Figure 7E

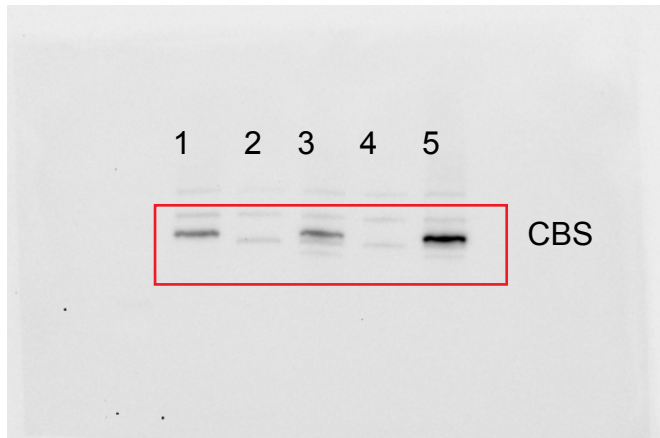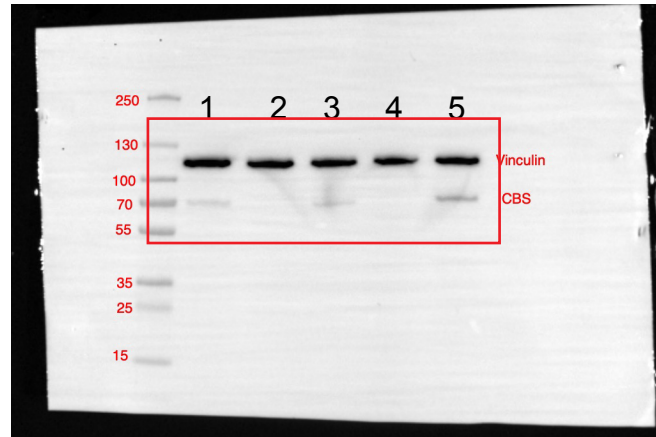

- 1 CBSWT DOX+
- 2 CBS WT DOX-
- 3 CBSI278T DOX+
- 4 CBS I278T DOX-
- 5 GES1

Supplement: Figure 7—source data 3. — Raw images were acquired using the ChemiDoc system (Bio-Rad). [file elife-71929-fig7-data3.pdf]
