# Supplementary material for: Cystathionine-β-synthase is essential for AKT-induced senescence and suppresses the development of gastric cancers with PI3K/AKT activation
Source: eLife. 2022 Jun 27;11:e71929. doi: 10.7554/eLife.71929 (PMC9236611; doi:10.7554/eLife.71929)
Supplement: Figure 2—source data 2. — Raw images were acquired using the ChemiDoc system (Bio-Rad). [file elife-71929-fig2-data2.pdf]

Figure 2-source data 2

Unedited immunoblots of Figure 2C

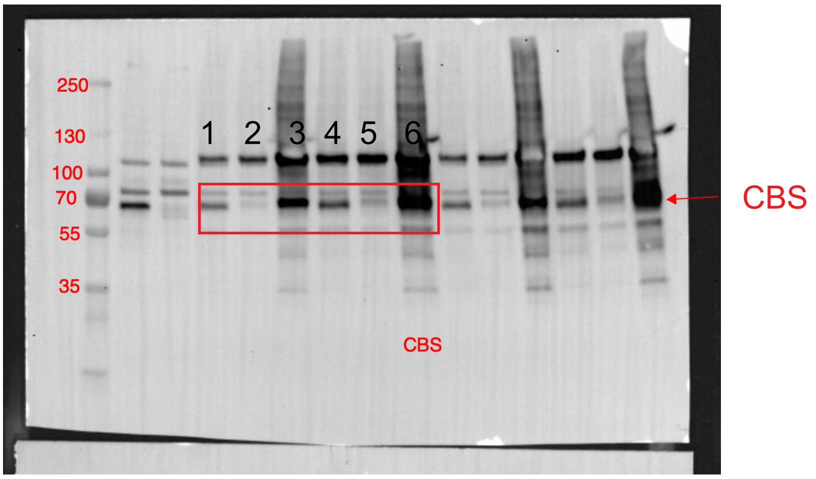

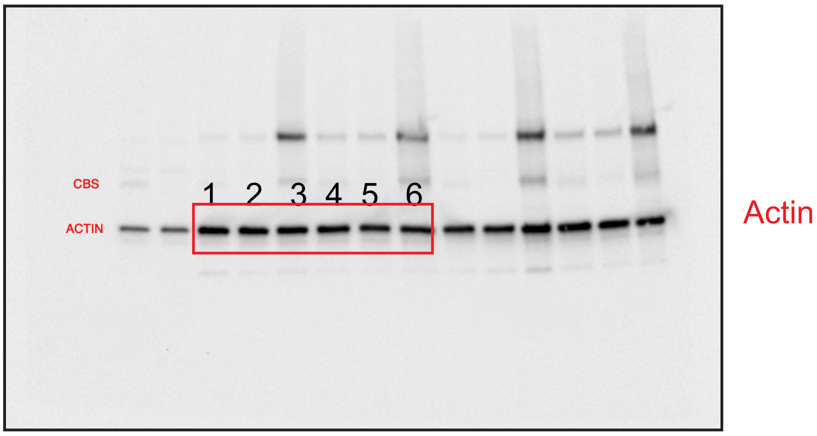

1. pBabe DOX- 4-OHT-
2. pBabe DOX+4-OHT-
3. pBabe DOX+ 4-OHT+
4. myrAKT1 DOX- 4-OHT-
5. myrAKT1 DOX+ 4-OHT-
6. myrAKT1 DOX+ 4-OHT+
