# Supplementary material for: Cystathionine-β-synthase is essential for AKT-induced senescence and suppresses the development of gastric cancers with PI3K/AKT activation
Source: eLife. 2022 Jun 27;11:e71929. doi: 10.7554/eLife.71929 (PMC9236611; doi:10.7554/eLife.71929)
Supplement: Figure 4—source data 3. — Raw images were acquired using the ChemiDoc system (Bio-Rad). [file elife-71929-fig4-data3.pdf]

Figure 4-source data 3

Unedited immunoblots of Figure 4F.

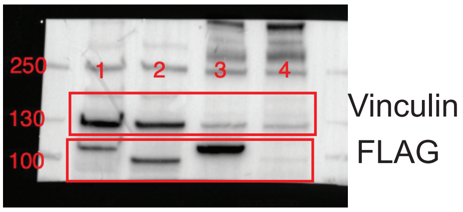

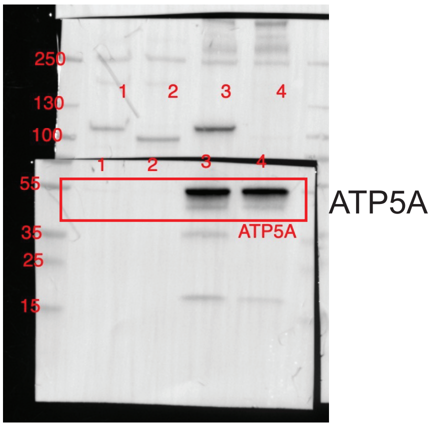

1. cytoplasm_WT-FLAG
2. Cytoplasm_Δ468-551
3. Mitohchondria_WT-FLAG
4. Mitochondria_Δ468-551
