# Supplementary material for: Cystathionine-β-synthase is essential for AKT-induced senescence and suppresses the development of gastric cancers with PI3K/AKT activation
Source: eLife. 2022 Jun 27;11:e71929. doi: 10.7554/eLife.71929 (PMC9236611; doi:10.7554/eLife.71929)
Supplement: Figure 5—figure supplement 1—source data 1. — Raw images were acquired using the ChemiDoc system (Bio-Rad). [file elife-71929-fig5-figsupp1-data1.pdf]

Figure 5-figure supplement 1-source data 1

Unedited immunoblots of Figure 5-figure supplement 1A

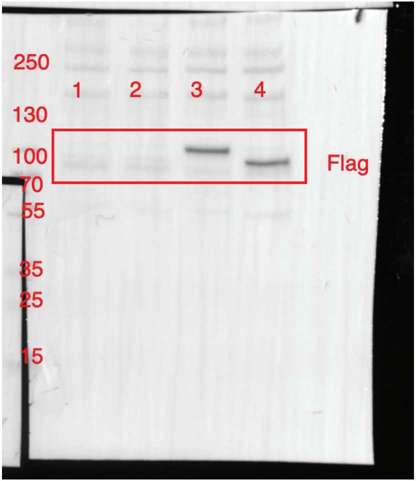

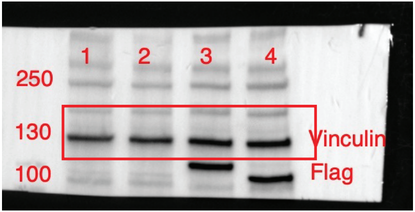

1 control
2. sh*CBS*
3.sh*CBS*_*CBS*WT
4. sh*CBS*_*CBS*Δ468-551
