# Supplementary material for: Cystathionine-β-synthase is essential for AKT-induced senescence and suppresses the development of gastric cancers with PI3K/AKT activation
Source: eLife. 2022 Jun 27;11:e71929. doi: 10.7554/eLife.71929 (PMC9236611; doi:10.7554/eLife.71929)

Figure 7-source data 2

Unedited immunoblots of Figure 7C

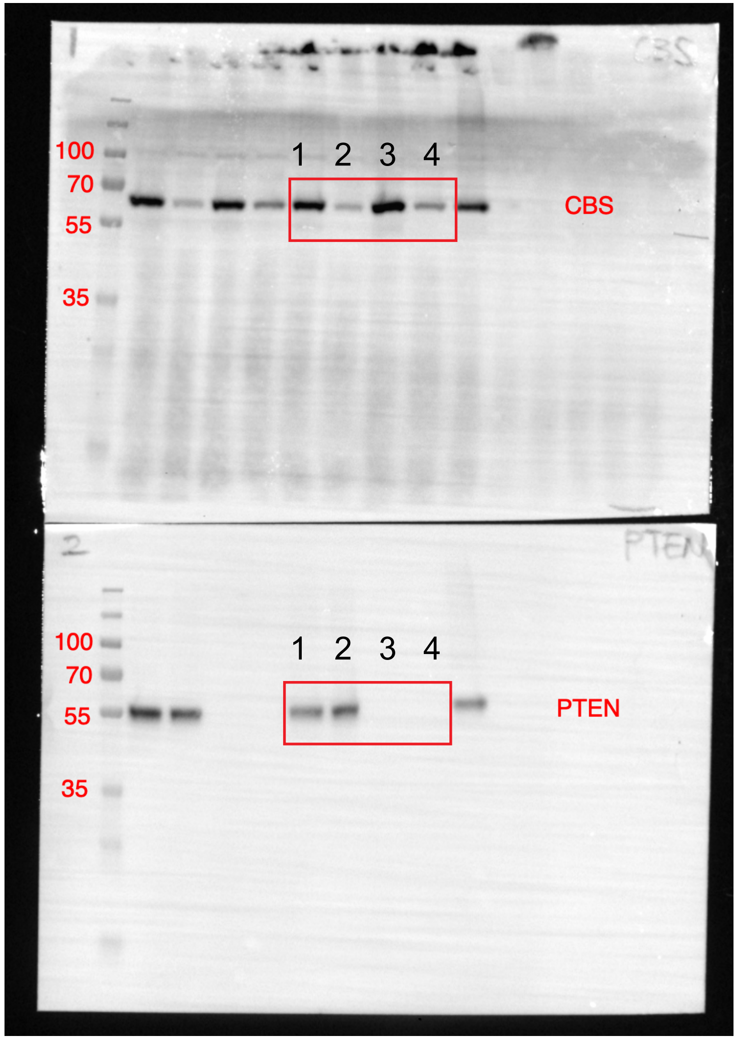

1. Cas9_sh*CBS*-
2. Cas9_sh*CBS*+
3. sg*PTEN*_sh*CBS*-
4. sg*PTEN*_sh*CBS*+

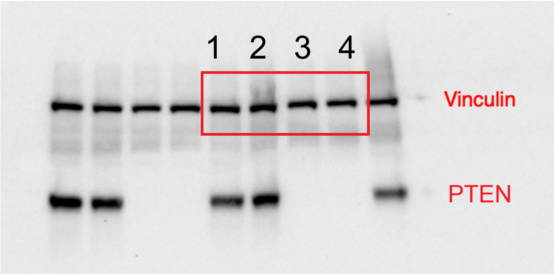

Supplement: Figure 7—source data 2. — Raw images were acquired using the ChemiDoc system (Bio-Rad). [file elife-71929-fig7-data2.pdf]
